# Association between active cytomegalovirus infection and lung fibroproliferation in adult patients with acute respiratory distress syndrome: a retrospective study

**DOI:** 10.1186/s12879-022-07747-y

**Published:** 2022-10-14

**Authors:** Zhihui Zhang, Rujian Li, Yubiao Chen, Jierong Zhang, Yongxin Zheng, Minmin Xu, Jiaqi Liang, Jiahui Li, Yongbo Huang, Yonghao Xu, Weiqun He, Xiaoqing Liu, Yimin Li

**Affiliations:** 1 Department of Critical Care Medicine, State Key Laboratory of Respiratory Diseases, National Clinical Research Center for Respiratory Disease, Guangzhou Institute of Respiratory Health, The First Affiliated Hospital of Guangzhou Medical University, Guangzhou, 510120 Guangdong People’s Republic of China; 2 Guangzhou Medical University, Guangzhou, 511436 Guangdong People’s Republic of China

**Keywords:** Cytomegalovirus, Acute respiratory distress syndrome, Fibroproliferation, High-resolution computer tomography, N-terminal peptide of serum procollagen III

## Abstract

**Background:**

Cytomegalovirus (CMV) has high seroprevalence, and its active infection is associated with several adverse prognoses in adult patients with acute respiratory distress syndrome (ARDS). However, the role of active CMV infection in ARDS-associated fibroproliferation is unknown. This study aimed at determining the association between active CMV infection and lung fibroproliferation in adult patients with ARDS.

**Methods:**

We retrospectively reviewed the medical records of all adult patients with ARDS who were admitted to the intensive care unit (ICU) from January 2018 to December 2020 at a national university-affiliated hospital in China. Study subjects were divided into active and non-active CMV infection groups based on CMV DNAemia within a 28-day ICU hospitalization. Lung fibroproliferation was measured using chest high-resolution computed tomography (HRCT) and N-terminal peptide of serum procollagen III (NT-PCP-III) within the first 28 days of ICU admission. Pulmonary fibrosis, clinical features, laboratory findings, treatment measures, and clinical outcomes were compared between the two groups.

**Results:**

Among the 87 ARDS patients included in this study, the incidence of active CMV infection was 16.1% within the 28-day ICU admission period. In logistic regression analyze, active CMV infection was found to be associated with higher pulmonary fibrogenesis, pulmonary fibrosis score, and NT-PCP-III level (*P* < 0.05). The duration of ICU stay in ARDS patients with active CMV infection was significantly higher than in those without active CMV infection (*P* < 0.05).

**Conclusions:**

Among adult patients with ARDS, active CMV infection was related to poor clinical outcomes. Active CMV infection was associated with ARDS-associated fibroproliferation. Prophylactic and preemptive use of anti-CMV agents on pulmonary fibrosis should be assessed to determine a consensus therapeutic strategy.

**Supplementary Information:**

The online version contains supplementary material available at 10.1186/s12879-022-07747-y.

## Introduction

Cytomegalovirus (CMV) is a latent infection virus that is widespread in the population [1]. CMV may reactivate under certain circumstances, and its hazardous nature has been proven, especially in immunosuppressed patients [2]. However, several studies have found that CMV reactivation also exists in immunocompetent patients with a critical illness [3]. Among those patients, the incidence of CMV reactivation, which is more than 30% is associated with prolonged length of mechanical ventilation, increased duration of hospital stay, and higher mortality [4].

The incidence of acute respiratory distress syndrome (ARDS) in the intensive care unit (ICU) is still higher than 10%, and the case fatality rate is even higher at 40% [5, 6]. Recent studies have shown that the incidence of CMV reactivation in ARDS patients is 22.0–27.3% and is also associated with adverse prognoses [7, 8]. Moreover, pulmonary fibrosis is one of the important reasons for the progressive aggravation of ARDS [9]. At present, in-vivo experiments and clinical research have preliminarily shown that active CMV infection induces pulmonary fibrogenesis, and the fibrogenesis may lead to the decline of pulmonary oxygenation among sepsis patients [10, 11]. However, in ARDS patients, the role of active CMV infection in lung fibroproliferation is not clear. Furthermore, chest high-resolution computed tomography (HRCT) and N-terminal peptide of serum procollagen III (NT-PCP-III) are considered reliable assessment methods for assessing ARDS-associated fibroproliferation [12, 13]. Therefore, this study aimed at investigating the association between active CMV infection and lung fibroproliferation in ARDS patients by chest HRCT and NT-PCP-III.

## Methods

### Patient screening

We retrospectively reviewed the medical records of all adult patients with ARDS who were admitted to the ICU between January 2018 and December 2020 at the First Affiliated Hospital of Guangzhou Medical University in China. CMV DNAemia was detected using real-time PCR for laboratory diagnosis of active CMV infection [14–16]. The study subjects were divided into active CMV infection (≧ 500 copies/mL) and non-active CMV infection groups (< 500 copies/mL) based on CMV DNAemia within a 28-day ICU hospitalization period. HRCT was recorded and NT-PCP-III was detected in the two groups at the set time interval. The Ethics Committees of the First Affiliated Hospital of Guangzhou Medical University approved the study protocol (No. GY-2021-K04).

### Data collection

The clinical data of 87 patients, including clinical features, laboratory findings, treatment measures, and clinical outcomes, were extracted from the electronic records by two independent intensivists who subsequently cross-checked their records for data accuracy. Furthermore, disagreements were further adjudicated by a third independent reviewer (an authoritative expert). Finally, all case data were entered into the electronic database for statistical analysis.

### Study definitions

The study met all of the following criteria: (1) the diagnostic criteria for ARDS (Berlin definition 2012) [9] within 24 h of ICU admission; (2) age > 18 years; (3) informed consent and retention of blood specimens; (4) chest HRCT was performed with the following intervals: admission to general ward ~ 28 days after ICU entry; (5) chest HRCT was performed ≧ 2 times; (6) interval between two chest HRCTs ≧ 7 days. Any of the following exclusion criteria was sufficient for exclusion: (1) pregnancy or lactation; (2) survival time < 72 h; (3) CMV not detected; (4) antiviral therapy before ICU admission; (5) presence of pulmonary fibrosis on the first chest HRCT.

CMV positive (active CMV infection) load values (CMV DNA) were calibrated to the HCMV World Health Organization Standard and relevant clinical study definitions, defined as a load greater than or equal to 500 copies/mL in plasma [14–16]. Included study subjects (87 cases) were tested for CMV at ICU admission, and tested for CMV at least once a week thereafter. Meanwhile, the management of those ARDS patients was strictly based on relevant treatment measures of international consensus [17], including low tidal volume ventilation, inspiratory pressure ventilation, prone positioning, etc.

### Assessment of pulmonary fibrosis

#### Chest HRCT

Chest HRCT was performed in the supine position, and image data acquisition was completed with spiral scanning for the study subjects. All chest HRCT scans were performed at the end of inspiration. Chest HRCT scans were performed from the lung apex to the diaphragmatic crest, slice thickness: 1.25 mm, pitch: 10 mm; scanning conditions were: 120 kV, 250 mA, images were displayed in the matrix: 512 × 512, slice thickness: 1.25 mm, and high spatial resolution reconstruction. All images were taken as lung window (window width 2000 Hu, window position 550 Hu) and mediastinal window (window width 450 Hu, window position 55 Hu). The extent of fibrotic lesions was observed under lung window conditions.

Subsequently, three radiologists read and scored images individually, and the resulting scoring results were averaged. Furthermore, a radiologist judged between ground glass, grid, honeycomb, and solid variants, according to the Fleischner Society definitions for HRCT terminology in the lung [18]. The lungs were divided into three levels according to their anatomical structure in six locations: lung apex to aortic arch level, aortic arch to inferior pulmonary vein level, and inferior pulmonary vein to lung base level. The pattern of signs of pulmonary fibrosis included simple ground-glass opacity, the mixture of ground-glass opacity and grid shadow, simple grid shadow, and honeycomb. We further quantified and defined imaging findings as follows: First, we defined the occurrence of pulmonary fibrosis as ‘1’ and the absence of pulmonary fibrosis as ‘0.’ Second, we referred to Prof. Ellen L Burnham’s method for scoring based on the area occupied by signs of pulmonary fibrosis [12]: the extent of involvement for each pattern was assigned a numerical score, where 0 = no involvement, 1 ≦ 5% involvement/minimal/not normal, 2 = 5–25% involvement, 3 = 26–49% involvement, 4 = 50–75% involvement, and 5 ≧ 75% involvement. Assessment of lung fibroproliferation by chest HRCT was completed within 28 days of ICU admission.

#### NT-PCP-III

A clinical blood sample resource bank was established at our medical center, and we routinely collected and stored patient blood specimens at regular intervals after admission to the ICU when the patients or their relatives signed the informed consent form. The study was approved by the ethics review board of the First Affiliated Hospital of Guangzhou Medical University.

Serum samples were collected from each ARDS patient at the time of enrollment (Day 1, 28). Refer to a similar approach by the Prof. Xu [19], the method of serum collection: whole blood was collected in procoagulant tubes and centrifuged at 2500 rpm for 10 min, and serum in the supernatant was collected and stored at − 80 °C until detection. Serum levels of NT-PCP-III (Day 1 and Day 28) were measured by enzyme-linked immunosorbent assay (ELISA) (SEA573Hu, USCN, Houston, TX, Lot: L210714899) according to the manufacturer’s instructions.

### Study outcomes

The primary outcomes of this study included the chest HRCT and NT-PCP-III differences between active and non-active CMV infection groups. Moreover, we also assessed secondary outcomes between the two groups, including clinical features, laboratory findings, treatment measures, and clinical outcomes.

### Statistical analysis

All statistical analyses or charting were performed using SPSS (SPSS Inc., USA, version 25.0) and GraphPad Prism (GraphpadSoftware Inc., USA, version 8.0). Continuous variables were expressed as Mean ± SD or Median (interquartile ranges, IQRs) and were compared with the Wilcoxon rank-sum test. Categorical variables were expressed as counts and percentages and were compared using the Chi-square test or Fisher’s exact test as appropriate. Logistic regression was used to determine the association between active CMV infection and pulmonary fibrosis indicators, including chest HRCT scores and NT-PCP-III level. Correlation coefficients between chest HRCT scores and NT-PCP-III level were calculated using the Spearman correlation coefficient. Additionally, risk factors for active CMV infection were screened with the univariate logistic regression and the variables with a *P* < 0.05 was considered as the potential risk factors, furthermore, the potential risk factors were imported into the multivariate logistic regression analysis. The regression coefficient (β), odds ratio (OR), and 95% confidence interval (CI) were calculated for establishing the active CMV infection risk model. The predictive value of active CMV infection was evaluated by using the receiver operating characteristic (ROC) curve. Moreover, we further calculated the area under the ROC curve (AUC), specificity, sensitivity, 95% CI, and P-value. The significance threshold was set at a two-sided *P* < 0.05.

## Results

### Active CMV infection

**Fig. 1:**
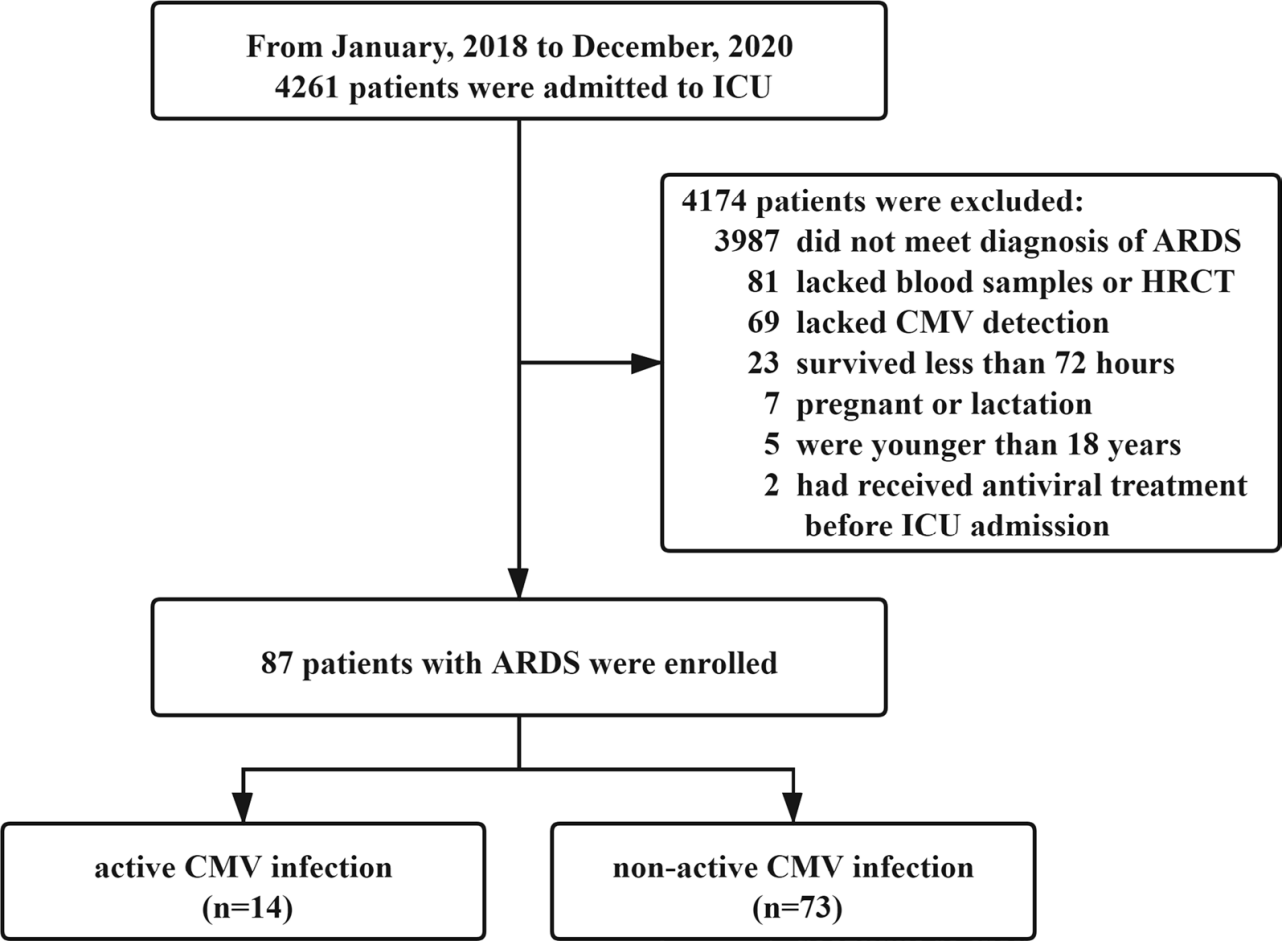
Flowchart for study subjects enrollment

From January 2018 to December 2020, a total of 4261 patients were transferred to the ICU. As shown in Fig. 1, 4174 cases were excluded because of a non-ARDS diagnosis (n = 3987), lack of blood samples or chest HRCT (n = 81), non-CMV detection (n = 69), death within 72 h after ICU admission (n = 23), pregnancy or lactation (n = 7), juveniles (n = 5), and receiving antiviral therapy before ICU entry (n = 2). Eventually, 87 cases were enrolled with complete data for analysis. Furthermore, 14 of 87 cases (16.1%) had active CMV infection; so, the study subjects were divided into an active CMV infection group (n = 14, 16.1%) and a non-active CMV infection group (n = 73, 83.9%).

### Patients’ features

The clinical features of 87 patients enrolled in the study are presented in Table 1. The study included 87 cases for statistical analysis, of whom 52 were male (59.8%). The mean age of the study population was 56 ± 16 years. Of the patients, 16 (18.4%) had mild ARDS, 40 (46.0%) had moderate ARDS, and 31 (35.6%) had severe ARDS. The patients’ condition was severe because the scores for APACHE II (median: 19) and SOFA (median: 9) were high. Severe pneumonia (94.3%) is the main cause of ARDS. The main comorbidities were cardiovascular diseases (n = 27, 31.0%), hypertension (n = 25, 28.7%), and diabetes (n = 15, 17.2%). Nevertheless, the differences in those clinical features between the two groups were not significant.

**Table 1:** Clinical features

Clinical features	Total	Active CMV infection		*P*
	N = 87	Yes (n = 14, 16.1%)	No (n = 73, 83.9%)	
Age (year)	56 ± 16	62 ± 11	54 ± 17	0.103
Sex, n (%)				0.331
Male	52 (59.8)	10 (71.4)	42 (57.5)	**–**
Female	35 (40.2)	4 (28.6)	31 (42.5)	**–**
Weight (kg)	60.0 (50.0–67.3)	60.0 (50.0–65.0)	60.0 (52.0–67.0)	0.939
BMI (kg/m^2^)	22.5 (19.6–24.2)	22.5 (18.5–23.1)	22.5 (19.7–24.2)	0.538
Score of disease severity				
APACHE II	19 (15–23)	19 (14–26)	19 (15–23)	0.689
SOFA	9 (6–11)	9 (6–13)	9 (7–11)	0.855
qSOFA	2 (1–3)	2 (2–3)	2 (1–3)	0.834
ARDS severity, n (%)				
Mild	16 (18.4)	1 (7.1)	15 (20.5)	0.418
Moderate	40 (46.0)	7 (50.0)	33 (45.2)	0.742
Severe	31 (35.6)	6 (42.9)	25 (34.3)	0.755
Main causes of ARDS, n (%)				0.588
Severe pneumonia	82 (94.3)	14 (100)	68 (93.2)	–
Other	5 (5.7)	0 (0)	5 (6.8)	–
Vital signs				
Average blood pressure (mmHg)	80 ± 22	83 ± 24	81 ± 19	0.449
Heart rate (bpm)	104 (86–120)	90 (83–122)	105 (87–120)	0.783
Respiratory rate (bpm)	24 (20–31)	24 (21–30)	24 (20–32)	0.913
Temperature (℃)	37.0 (36.6–38.0)	37.1 (36.8–38.2)	37.0 (36.6–37.9)	0.452
Comorbidities, n (%)				
Cardiovascular diseases	27 (31.0)	5 (35.7)	22 (30.1)	0.922
Hypertension	25 (28.7)	7 (50.0)	18 (24.7)	0.110
Diabetes	15 (17.2)	3 (21.4)	12 (16.4)	0.947
COPD	13 (14.9)	2 (14.3)	11 (15.1)	> 0.999
Connective tissue disease*	12 (13.8)	2 (14.3)	10 (13.7)	> 0.999
Malignancy	12 (13.8)	2 (14.3)	10 (13.7)	> 0.999
Cerebral thrombosis	8 (9.2)	2 (14.3)	6 (8.2)	0.830

CMV: Cytomegalovirus; BMI: Body Mass Index; APACHE II: Acute Physiology and Chronic Health Evaluation II; qSOFA: Quick Sequential Organ Failure Assessment; ARDS: acute respiratory distress syndrome; CAP: community-acquired pneumonia; HAP: hospital-acquired pneumonia; COPD: chronic obstructive pulmonary disease

Categorical variables were expressed as n (%), Continuous variables were expressed as Mean ± SD or Median (IQRs); ^*^, included Systemic Lupus Erythematosus, rheumatoid arthritis, Dermatomyositis, Still’s Disease and Sjogren’s Syndrome. Bold font indicates the difference was statistically significant

Furthermore, laboratory findings of the study subjects at ICU admission are presented in Table 2. The active CMV infection group showed lower platelet counts [median: 71 vs. 151 (10^9^/L), *P* = 0.013] and NK cell proportion [median: 4.7 vs. 11.9 (%), *P* = 0.030] than the non-active CMV infection group. There were, however, no significant differences in other laboratory findings between the two groups.

**Table 2:** Laboratory findings at ICU admission

Laboratory findings	Total	Active CMV infection		*P*
	N = 87	Yes (n = 14, 16.1%)	No (n = 73, 83.9%)	
Oxygenation (P/F)	126 (79–183)	118 (77–171)	128 (80–185)	0.524
White blood cells (10^9^/L)	11.3 (7.5–15.0)	9.7 (6.9–14.0)	11.6 (7.6–15.1)	0.407
Neutrophils (10^9^/L)	10.6 (6.6–14.0)	8.9 (6.6–12.2)	10.8 (6.7–14.4)	0.538
Lymphocytes (10^9^/L)	0.4 (0.2–0.7)	0.4 (0.1–0.5)	0.4 (0.2–0.8)	0.224
Monocytes (10^9^/L)	0.5 (0.2–1.0)	0.4 (0.1–0.6)	0.6 (0.2–1.1)	0.115
Hemoglobin (g/L)	93 (79–108)	84 (78–95)	96 (81–112)	0.072
**Platelet**^**a**^** (10**^**9**^**/L)**	**137 (79–221)**	**71 (44–136)**	**151 (94–232)**	**0.013**
Procalcitonin (ng/mL)	1.13 (0.28–4.89)	1.56 (0.41–13.99)	1.06 (0.28–4.69)	0.490
Blood lactate (mmol/L)	1.89 (1.38–2.73)	1.81 (1.36–2.23)	1.92 (1.40–2.81)	0.511
PT (s)	15.7 (15.0–17.6)	16.3 (15.4–17.8)	15.7 (14.9–17.3)	0.569
APTT (s)	42.5 (37.9–51.4)	45.5 (37.9–65.5)	42.2 (37.9–49.1)	0.188
Cardiac troponins (ng/mL)	0.06 (0.03–0.41)	0.10 (0.05–0.14)	0.06 (0.03–1.10)	0.704
NT-proBNP (pg/mL)	1738 (528–4284)	2522 (660–7487)	1718 (453–3054)	0.167
AST (U/L)	48.7 (33.1–81.9)	43.9 (38.0–62.0)	48.7 (32.7–89.0)	0.448
ALT (U/L)	26.7 (15.4–56.6)	25.8 (18.9–43.4)	27.7 (15.4–58.5)	0.352
Albumin (g/L)	31.4 (27.8–34.7)	32.3 (27.8–34.9)	31.1 (27.8–34.5)	0.918
T-BIL (μmol/L)	15.2 (9.5–25.0)	21.1 (9.5–26.5)	15.0 (9.7–20.5)	0.150
D-BIL (μmol/L)	5.0 (2.5–11.5)	8.6 (2.1–11.3)	4.8 (2.7–12.2)	0.388
Scr (μmol/L)	102 (72–177)	173 (77–270)	99 (72–163)	0.184
BUN (mmol/L)	11.0 (6.7–17.8)	13.0 (7.2–34.4)	10.8 (6.6–17.3)	0.194
T lymphocytes (%)	66.5 (57.1–76.8)	64.6 (58.3–72.1)	67.6 (58.2–73.5)	0.885
Th lymphocytes (%)	34.9 (25.2–46.1)	35.6 (27.0–37.3)	32.5 (25.3–41.5)	0.391
Ts lymphocytes (%)	24.5 (17.1–33.2)	23.1 (19.6–30.5)	28.8 (19.6–33.2)	0.287
Th/Ts	1.22 (0.97–2.24)	1.36 (1.01–1.58)	1.15 (0.96–1.78)	0.394
B lymphocytes (%)	17.7 (7.6–26.5)	13.1 (1.1–32.4)	17.5 (12.9–24.9)	0.606
**NK cells**^**a**^** (%)**	**9.6 (6.0–15.9)**	**4.7 (2.6–7.4)**	**11.9 (7.0–15.7)**	**0.030**
IL-2 (pg/mL)	1.00 (0.60–1.36)	0.97 (0.52–1.34)	1.00 (0.67–1.31)	0.872
IL-4 (pg/mL)	1.41 (0.84–2.43)	0.93 (0.74–1.78)	1.51 (0.91–2.23)	0.439
IL-6 (pg/mL)	55.86 (12.80–181.87)	88.42 (32.83–153.56)	51.95 (12.77–168.13)	0.651
IL-10 (pg/mL)	6.03 (3.67–11.61)	7.00 (3.26–17.83)	5.31 (3.77–11.28)	0.849
TNF-α (pg/mL)	1.01 (0.74–1.65)	0.94 (0.59–1.30)	1.06 (0.76–1.65)	0.350
INF-γ (pg/mL)	1.38 (0.69–3.68)	1.21 (0.64–6.01)	1.41 (0.74–3.51)	0.895

CMV: Cytomegalovirus; P/F: PaO_2_/FiO_2_; PT: prothrombin time; APTT: activated partial thromboplastin time; NT-proBNP: N-terminus Precursor of B-type Natriuretic Peptide; AST: aspartate aminotransferase; ALT: alanine transaminase; T-BIL: total bilirubin; D-BIL: direct bilirubin; Scr: serum creatinine; BUN: serum urea nitrogen; Th: T-helper lymphocytes; Ts: T-suppressor Lymphocytes; NK: natural killer; IL: Interleukin; TNF: Tumor necrosis factor; INF: Interferon

^a^P < 0.05; Continuous variables were expressed as Median (IQRs); Bold font indicates the difference was statistically significant

### Pulmonary fibrosis

**Fig. 2:**
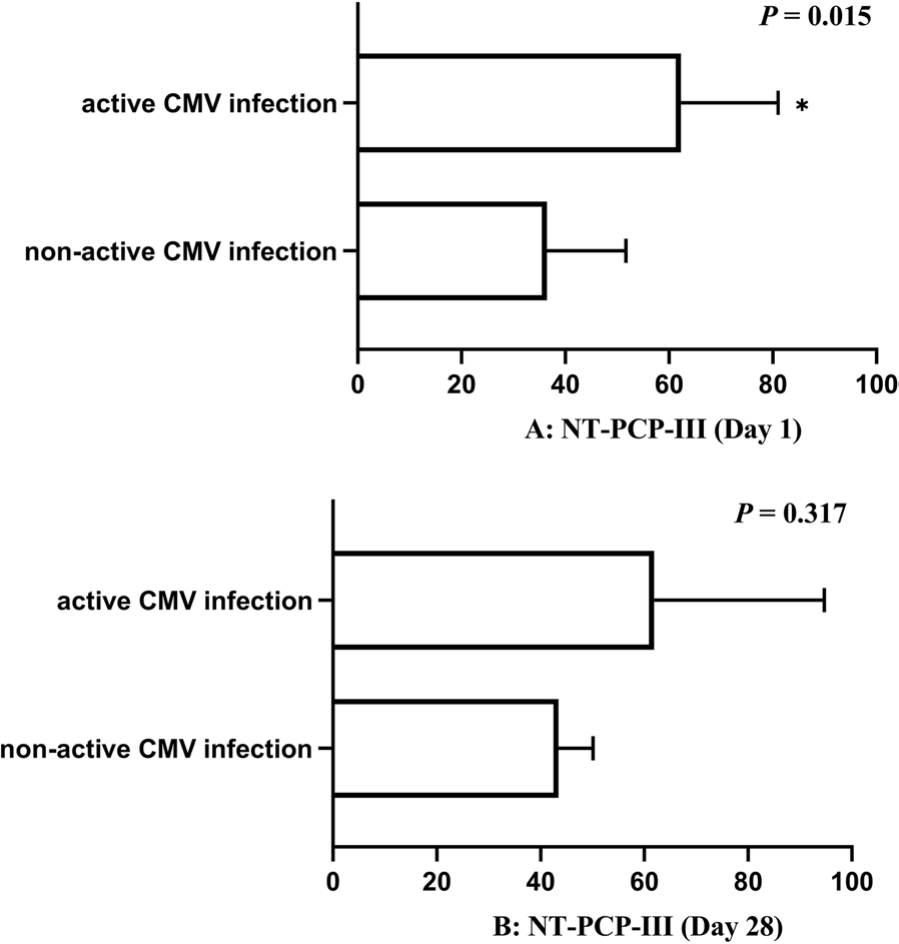
**A**, **B** Assessment of pulmonary fibrosis by NT-PCP-III. *P < 0.05; Continuous variables (Plot) were expressed as Median (interquartile ranges)

The association between active CMV infection and lung fibroproliferation is shown in Table 3, Additional file 1: Table S1, and Fig. 2. Pulmonary fibrogenesis [64.3% vs. 34.3% (n), *P* = 0.035], pulmonary fibrosis score [median: 1 vs. 0 (d), *P* = 0.031], and NT-PCP-III level (Day 1) [median: 62.3 vs. 36.5 (ng/mL), *P* = 0.015] in patients with active CMV infection were significantly higher than in those without active CMV infection. The level of NT-PCP-III (Day 28) [median: 61.9 vs. 43.8 (ng/mL), *P* = 0.317] was high in cases with active CMV infection than in cases with non-active CMV infection, although statistical significance was not reached. In the logistic regression analyses, higher pulmonary fibrogenesis [β: 1.240, OR: 3.456, 95% CI 1.046–11.421, *P* = 0.042], pulmonary fibrosis score [β: 0.895, OR: 2.447, 95% CI 1.042–5.748, *P* = 0.040], and NT-PCP-III level (Day 1) [β: 0.018, OR: 1.019, 95% CI 1.001–1.036, *P* = 0.038] were found to be associated in patients with active CMV infection. Furthermore, the Spearman analysis revealed strong correlation [r = 0.249, *P* = 0.020] between chest HRCT score and NT-PCP-III (Day 1), as shown in Additional file 1: Table S2.

**Table 3:** Association between active CMV infection and pulmonary fibrosis indicators

Variables	Active CMV infection			
	β	OR	95% CI	*P*
Chest HRCT				
Pulmonary fibrogenesis^a^	**1.240**	**3.456**	**1.046–11.421**	**0.042**
Pulmonary fibrosis score^a^	**0.895**	**2.447**	**1.042–5.748**	**0.040**
NT-PCP-III				
Day 1^a^	**0.018**	**1.019**	**1.001–1.036**	**0.038**
Day 28	0.024	1.024	0.991–1.059	0.153

CMV: Cytomegalovirus; HRCT: high-resolution computed tomography; NT-PCP-III: N-terminal Peptide of Serum Procollagen III; β: regression coefficient; OR: odds ratio; CI: confidence interval

^a^P < 0.05; Bold font indicates the difference was statistically significant

### Treatment measures, complications, and clinical outcomes

It is clear from Table 4 that significantly more patients with active CMV infection underwent blood transfusion [35.7% vs. 11.0% (n), *P* = 0.049] and antiviral therapy [100% vs. 0% (n), *P* < 0.001] than patients with non-active CMV infection.

**Table 4:** Treatment measures, complications, and clinical outcomes

	Total	Active CMV infection		*P*
	N = 87	Yes (n = 14, 16.1%)	No (n = 73, 83.9%)	
Treatment measures, n (%)				
Before ICU admission				
Glucocorticoids	32 (36.8)	6 (42.9)	26 (35.6)	0.607
Immunosuppressive drugs^*^*^	12 (13.8)	2 (14.3)	10 (13.7)	> 0.999
Gamma globulin infusions	10 (11.5)	0 (0)	10 (13.7)	0.310
Blood transfusion^a^	**13 (15.0)**	**5 (35.7)**	**8 (11.0)**	**0.049**
After ICU admission				
Glucocorticoids	25 (28.7)	3 (21.4)	22 (30.1)	0.736
Immunosuppressive drugs^*^*^	4 (4.6)	1 (7.1)	3 (4.1)	0.511
Gamma globulin infusions	19 (21.8)	4 (28.6)	15 (20.6)	0.755
Blood transfusion	38 (43.7)	8 (57.1)	30 (41.1)	0.267
Antiviral therapy^*#*a^	**14 (16.1)**	**14 (100)**	**0 (0)**	**< 0.001**
CRRT	34 (39.1)	7 (50.0)	27 (37.0)	0.361
ECMO	13 (14.9)	2 (14.3)	11 (15.1)	> 0.999
Complications, n (%)				
Septic shock^a^	**56 (64.4)**	**13 (92.9)**	**43 (58.9)**	**0.034**
AKI	44 (50.6)	10 (71.4)	34 (46.6)	0.088
DIC	24 (27.6)	6 (42.9)	18 (24.7)	0.285
AHF	16 (18.4)	5 (35.7)	11 (15.1)	0.147
AECOPD	13 (14.9)	2 (14.3)	11 (15.1)	> 0.999
Clinical outcomes				
28-day VFD (d)	0 (0–13)	0 (0–0)	0 (0–14)	0.134
Length of IMV	27 (13–55)	40 (26–82)	24 (13–51)	0.072
Duration of ICU stay^a^ (d)	**30 (15–57)**	**51 (26–104)**	**29 (15–57)**	**0.035**
Duration of hospital stay (d)	39 (26–85)	56 (32–127)	39 (26–80)	0.330
In-hospital mortality, n (%)	8 (9.2)	2 (14.3)	6 (8.2)	0.830

CMV: Cytomegalovirus; CRRT: continuous renal replacement therapy; ECMO: extracorporeal membrane oxygenation; AHF: acute heart failure; AKI: acute kidney failure; DIC: disseminated intravascular coagulation; VFD: ventilator-free days; IMV: invasive mechanical ventilation

^a^P < 0.05; Categorical variables were expressed as n (%), Continuous variables were expressed as Median (IQRs); ^#^, included ganciclovir, valganciclovir, or sodium phosphate; ^^^, included cyclophosphamide, methotrexate, and mycophenolate mofetil. Bold font indicates the difference was statistically significant

Moreover, compared to the non-active CMV infection group, the number of ARDS patients with septic shock complications [92.9% vs. 58.9% (n), *P* = 0.034] was higher in the active CMV infection group. Nevertheless, there were no statistically significant differences for other treatment measures and complications between the two groups.

As shown in Additional file 1: Table S3, in the multivariate regression model, platelet [OR: 0.991, 95% CI 0.983–1.000, *P* = 0.042] and septic shock [OR: 9.097, 95% CI 1.097–75.453, *P* = 0.041] were independently associated with active CMV infection in ARDS patients. Based on the regression coefficient (β), platelet [β: − 0.009] was an inhibitory factor and septic shock [β: 2.208] was a promotive factor for active CMV infection. We further plotted ROC curves for platelet to assess the active CMV infection predictive value. From Additional file 1: Table S4, it can be seen that the AUC of platelet was 0.711 [Specificity: 88.7%, Sensitivity: 50.0%; 95% CI 0.562–0.860; *P* = 0.013], so platelet had a moderate predictive value for active CMV infection. The pathogens caused by ARDS is shown in Additional file 1: Table S5, but the differences in those pathogens between the two groups were not significant. And the detection time-point of CMV DNAemia and chest HRCT Scans are shown in Additional file 1: Table S6.

From Table 4, the duration of ICU stay [median: 51 vs. 29 (d), *P* = 0.035] was significantly higher in the active CMV infection group than in the non-active CMV infection group. Although statistical significance was not reached, the duration of hospital stay [median: 56 vs. 39 (d), *P* = 0.330], length of invasive mechanical ventilation (IMV) [median: 40 vs. 24 (d), *P* = 0.072], and the in-hospital mortality rate [14.3% vs. 8.2% (n), *P* = 0.830] were lower in cases without active CMV infection than in cases with active CMV infection.

## Discussion

This study aimed at determining the association between active CMV infection and lung fibroproliferation in adult patients with ARDS. Among adult patients with ARDS, the incidence of active CMV infection was 16.1%. In addition, clinical characteristics, including laboratory findings (lower level of platelet and NK cells), blood transfusion, and septic shock, were related to active CMV infection. Most importantly, further correlation statistical analysis revealed that active CMV infection was associated with pulmonary fibrosis based on chest HRCT and NT-PCP-III. Additionally, active CMV infection was associated with several adverse prognoses.

Active CMV infection is not an uncommon phenomenon in critically ill patients. Our research team’s systematic review and meta-analysis have shown that the incidence of reactivation in immunocompetent patients is up to 31% in critical care settings [4]. In a prospective, multicenter epidemiological study that enrolled 399 patients with ARDS, 271 (68%) cases were CMV seropositive, and reactivation occurred in 74 (27%) of them; so the incidence of active CMV infection was 18.6% [7]. An additional study, which enrolled 123 ARDS patients receiving extracorporeal membrane oxygenation (ECMO), revealed that CMV DNAemia occurred at any level in 22.0% of the patients [8]. Due to the different inclusion and exclusion criteria, diagnostic methods, and specimen detection, the above incidence was slightly different from the results of this study. In particular, the detection methods (time points and monitoring periods) of CMV in different studies may have an effect on the incidence of active CMV infection. The present study was retrospective and could not detect each time point clearly; so, the incidence of active CMV infection may be lower than the real situation. It is also essential to note that our study used blood DNAemia for evaluating active CMV infection, which might underestimate the active CMV infection, combined with a more comprehensive detection of lower respiratory tract specimens.

Previous studies have verified that low-level platelet and NK cells, blood transfusion, and septic shock are associated with active CMV infection [3, 20, 21], consistent with this study's results. The reason behind these associations is related to the direct pathological damage and indirect damage.

The inhibition of platelet and NK cells may be related to the damage caused by CMV infection to the hematopoiesis and immune system [21–23]. Specifically, for bone marrow transplantation patients, CMV infection causes delayed platelet recovery [23]. CMV infection can inhibit NK cells through driving adaptive epigenetic diversification with altered signaling and effector function [21, 24]. Moreover, active CMV infection through blood transfusion is a risk in treating patients with a critical illness. A study has indicated that donations from new CMV-IgG-positive donors bear the highest risk for transmitting CMV infections because they contain elevated CMV-DNA levels, a risk factor for active CMV infection [25]. Several studies have found that sepsis is associated with active CMV infection [10, 19, 26, 27]. An in vivo model has shown that inflammatory factors such as lipopolysaccharide (LPS), tumor necrosis factor-alpha (TNF-α), and interleukin-1beta (IL-1β) are associated with sepsis and could activate CMV immediate early genes and promote viral replication [28]. From clinical studies, it has also been speculated that CMV reactivation is associated with multiple inflammatory factors (IL-6 and TNF-α) [29, 30]. There was an interaction between septic shock and active CMV infection. However, there were cases of prolonged mechanical ventilation causeing ventilator-acquired pneumonia clinically, which might also lead to sepsis-related active CMV infection, though no statistically significant difference was reached between these pathogens and active CMV infection. Therefore, the limited sample size and unmeasured confounders were the inadequacies of this study.

Furthermore, our results showed that septic shock and platelet were independent risk factors for active CMV infection and were independent of each other in the study subjects. It was further found that platelet levels had a moderate value for predicting active CMV infection. The mechanisms through which septic shock causes active CMV infection have been described above, and this result was consistent with most of the current studies. Nonetheless, no relevant study is consistent with our results, and the association between CMV and platelet has never been shown before. Platelets are small masses of cytoplasm released from the cytoplasm of mature megakaryocytes in the bone marrow and are extremely important for the body’s hemostatic function. However, the latest view considers that platelets can be an important mediator of immunity and inflammation [31]. In allogeneic bone marrow transplantation recipients, platelet recovery was significantly slower in CMV-positive patients than in CMV-negative patients, the condition may be related to CMV-associated myelosuppression [23]. Evidence for the association between CMV and myelosuppression has been provided from in vitro/vivo studies and clinical studies, those results indicated the direct infection of hematopoietic progenitors and that the supportive microenvironment is infected, thereby compromising its supportive function [32]. Importantly, more patients need to be included for further confirmation of the role of platelets. Meanwhile, it needs to be emphasized that the relatively small sample size in this study opens the possibility for a type II error.

Current treatment protocols for ARDS continue to evolve and be refined; nevertheless, its incidence and case fatality remain high [5, 6]. Pulmonary fibrosis is a common complication of ARDS, and its severity significantly influences clinical outcomes and long-term quality of life in ARDS patients [33]. Multiple factors have been associated with ARDS-associated fibroproliferation, in which viral infection plays an important role, but the role of CMV infection in this is not definite. At present, in vitro results have shown that CMV from latent infection in the lung could undergo reactivation under LPS intervention, and active CMV infection could lead to the development of fibrosis in the lungs of mice [11]. Besides, a clinical study showed that active CMV infection could influence the oxygenation function (PaO_2_/FiO_2_) of critically ill patients with sepsis [10]. In summary, we hypothesized that active CMV infection was associated with pulmonary fibrosis leading to low oxygenation levels in ARDS patients; so, we further designed and carried out this study. There is no relevant clinical research consistent with our results, and the association between active CMV infection and lung fibroproliferation in adult patients with ARDS has never been studied before. We found that active CMV infection was associated with ARDS-associated fibroproliferation based on chest HRCT and NT-PCP-III. The active CMV infection group had a lower oxygenation level than the non-active CMV infection group, although statistical differences have not been observed. This revealed that CMV could damage the respiratory barrier, leading to pulmonary fibrosis and subsequently to decreased oxygenation. There is not well-established method to assess CT image of ARDS associated the lung fibrosis, thus in the study, we adapted the scoring system of CT image which is commonly used in evaluation of pulmonary fibrosis which is characterized by honeycombing. In this study, ARDS patients do not show typical honeycombing on HRCT scans. Meanwhile, pulmonary fibrosis once present in HRCT was largely refractory to disappear, so excluded patients with pulmonary fibrosis at the first HRCT scan who had excluded pre-existing pulmonary fibrosis. Moreover, the level of NT-PCP-III (Day 28) was high in cases with active CMV infection than in cases with non-active CMV infection, although statistical significance is not reached. Only 38 patients (6 in active CMV infection group and 32 in non-active group) still stayed in ICU on day 28 and NT-PCP-III were evaluated in these patients. The relatively small sample may be the potential reason for negative correlation between active CMV infection and NT-PCP-III at day 28. Furthermore, antiviral therapy was used in the active CMV infection group which effectively in suppressing the virus and reduced pulmonary fibroproliferation.

In addition, our assessment of pulmonary fibrosis using chest HRCT and NT-PCP-III is clinically feasible; both of these are reliable diagnostic methods or markers for ARDS-associated lung fibroproliferation [12, 13, 34], with the advantages of non-invasiveness and high specificity. Our results also found that chest HRCT score and NT-PCP-III had a strong correlation. Although lung histopathology is the gold standard, performing open lung biopsies in ARDS patients is complicated. Besides, the testing time points for chest HRCT were not consistent, and the endpoints were 28 days after admission to the ICU, and testing samples for NT-PCP-III were sera, which are slightly less sensitive relative to lower respiratory specimens (endotracheal aspirate or bronchoalveolar lavage fluid). Thus, the incidence of pulmonary fibrosis may have been underestimated. Furthermore, the source of fibrosis is also a matter of concern. Studies have shown that CMV infection could lead to epithelial–mesenchymal transition (EMT) in various cells, and continuous EMT is closely related to organ fibrosis [35–37]. EMT refers to the biological process in which epithelial cells lose epithelial characteristics and obtain mesenchymal characteristics under some physiological or pathological conditions [38]. EMT is transient and reversible, depending on its biological and functional environment, so preventive or preemptive anti-CMV treatment may benefit ARDS patients. In addition, the current studies have revealed that some patients occurred pulmonary fibrosis after COVID-19 pulmonary infection owing to SARS-CoV-2 infection promoted the occurrence of lung fibroproliferation [39, 40]. Due to the outbreak control requirements of the Chinese government, all patients with confirmed COVID-19 diseases were sent to the specialized infectious disease hospital or department for isolation and treatment. Therefore, no patients with COVID-19 pulmonary infection were included during this study period. Hemopexin (hx) level of pulmonary disease was associated with lung fibroproliferation [41, 42], but the study lacked detection of hx.

Most studies have shown that active CMV infection was associated with several poor clinical outcomes, which is consistent with our results, including prolonged duration of mechanical ventilation, hospitalization and ECMO, and increased complications and mortality [2–4, 7, 8, 10, 20, 23, 26–30]. The mechanisms include direct CMV pathogenicity or indirect CMV effects such as CMV-mediated immunosuppression and CMV-mediated lung injury [29, 43]. Moreover, the cause of death in both groups was multiple organ dysfunction syndrome in this study. Furthermore, the main cause of death in the active CMV infection group was multiorgan failure. As a matter of fact, it is very hard to tell the exact attribution to death when the patient is being in the end-stage of ARDS in clinical practice. In most cases, we believe that above conditions may contribute to the death of ARDS patients, the attribution of pulmonary fibroproliferation to death of ARDS patients need to be evaluated in a prospective well controlled study. In addition, the notable role of antiviral therapy in active CMV infection is well established. However, the preemptive and prophylactic application of anti-CMV therapy remains controversial for non-immunosuppressed patients [30, 44, 45], and the role of preemptive and prophylactic anti-CMV therapy in pulmonary fibrosis, which is not clear, should be further investigated in future studies.

This study has several limitations. First, it was a single-center retrospective study, so the time points at which CMV detections and chest HRCT scans were made were difficult to be determined. Therefore, the timing of when CMV was detected relative to admission and relative to HRCT scans might affect assessment of the association between active CMV infection and lung fibroproliferation. Second, only active infection, and not reactivation, could be assessed because of the lack of detection of CMV IgG. Third, the number of patients included was relatively insufficient to comprehensively evaluate the epidemiological characteristics and lung fibroproliferation of CMV for adult patients with ARDS. Fourth, due to the lack of CMV and NT-PCP-III detection in the lower respiratory tract, we could not further assess the association between CMV and NT-PCP-III among ARDS patients. Therefore, a prospective, multicenter study is needed in the future, and more study participants with ARDS should be included. Moreover, the effect of prophylactic and preemptive therapy using anti-CMV agents for pulmonary fibrosis should be evaluated in ARDS patients. Eventually, further assessment of the effect of CMV in the airways of ARDS-associated fibroproliferation markers is urgently needed.

## Conclusions

The incidence of active CMV infection was 16.1% in adult patients with ARDS. Active CMV infection was related to adverse clinical outcomes, including prolonged ICU hospitalization. Moreover, active CMV infection was associated with ARDS-associated fibroproliferation based on chest HRCT and NT-PCP-III. Therefore, further evaluation of the prophylactic and preemptive therapy using anti-CMV agents for pulmonary fibrosis should be conducted in the future to identify a consistent treatment strategy.

## Supplementary Information

**Additional file 1: Table S1.** Assessment of pulmonary fibrosis by HRCT and NT-PCP-III. **Table S2.** Correlation analysis of chest HRCT Score and NT-PCP-III. **Table S3.** Risk factors for active CMV infection. **Table S4.** Predictive value of platelet on active CMV infection evaluated by ROC. **Table S5.** Bacterial and fungal species. **Table S6.** Detection Time-point of CMV DNAemia and Chest HRCT Scans.

## Data Availability

The datasets generated during and analyzed during the current study are not publicly available due to patients privacy but are available from the corresponding author on reasonable request.

## Abbreviations

CMV: Cytomegalovirus
ICU: Intensive care unit
ARDS: Acute respiratory distress syndrome
HRCT: High-resolution computed tomography
NT-PCP-III: N-terminal peptide of serum procollagen III
MV: Mechanical ventilation
IMV: Invasive mechanical ventilation
BMI: Body Mass Index
APACHE II: Acute Physiology and Chronic Health Evaluation II
SOFA: Sequential Organ Failure Assessment
qSOFA: Quick Sequential Organ Failure Assessment
CAP: Community-acquired pneumonia
HAP: Hospital-acquired pneumonia
P/F: PaO_2_/FiO_2_
QRT-PCR: Quantitative real-time polymerase chain reaction
COPD: Chronic obstructive pulmonary disease
PT: Prothrombin time
APTT: Activated partial thromboplastin time
NT-proBNP: N-terminal Pro-B-type Natriuretic Peptide
AST: Aspartate aminotransferase
ALT: Alanine transaminase
T-BIL: Total bilirubin
Scr: Serum creatinine
BUN: Serum urea nitrogen
Hb: Hemoglobin
Th: T-helper lymphocytes
Ts: T-suppressor lymphocytes
NK: Natural killer
IL: Interleukin
TNF: Tumor necrosis factor
INF: Interferon
CRRT: Continuous renal replacement therapy
ECMO: Extracorporeal membrane oxygenation
AKI: Acute kidney injury
DIC: Disseminated intravascular coagulation
AHF: Acute heart failure
AECOPD: Acute exacerbation of chronic obstructive pulmonary disease
VFD: Ventilator-free days
IQRs: Interquartile ranges
r: Correlation coefficient
β: Regression coefficient
OR: Odds ratio
ROC: Receiver operating characteristic
AUC: Area under the ROC curve
CI: 95% Confidence interval
LPS: Lipopolysaccharide
EMT: Epithelial mesenchymal transition
Hx: Hemopexin
ETA: Endotracheal aspirates
G^+^: Gram-positive
G^−^: Gram-negative
