# Supplementary material for: Association between active cytomegalovirus infection and lung fibroproliferation in adult patients with acute respiratory distress syndrome: a retrospective study
Source: BMC Infect Dis. 2022 Oct 14;22:788. doi: 10.1186/s12879-022-07747-y (PMC9562065; doi:10.1186/s12879-022-07747-y)
Supplement: Supplementary file 1 — Additional file 1: Table S1. Assessment of pulmonary fibrosis by HRCT and NT-PCP-III. Table S2. Correlation analysis of chest HRCT Score and NT-PCP-III. Table S3. Risk factors for active CMV infection. Table S4. Predictive value of platelet on active CMV infection evaluated by ROC. Table S5. Bacterial and fungal species. Table S6. Detection Time-point of CMV DNAemia and Chest HRCT Scans. [file 12879_2022_7747_MOESM1_ESM.doc]

**Additional file (Tables)**

**Association between Active Cytomegalovirus Infection and Lung Fibroproliferation in Adult Patients with Acute Respiratory Distress Syndrome**

**Tables**

**Table S1. Assessment of Pulmonary Fibrosis by HRCT and NT-PCP-III.**

|  | **Total** | **Active CMV infection** | |  |
| --- | --- | --- | --- | --- |
|  | **N=87** | **Yes**  **(n=14, 16.1%)** | **No**  **(n=73, 83.9%)** | ***P*** |
| **HRCT** |  |  |  |  |
| **Pulmonary fibrogenesis^a^, n (%)** | **34 (39.1)** | **9 (64.3)** | **25 (34.3)** | **0.035** |
| **Pulmonary fibrosis** **score^a^** | **0 (0-1)** | **1 (0-1)** | **0 (0-1)** | **0.031** |
| **NT-PCP-III (ng/ml)** |  |  |  |  |
| **Day 1^a^** | **38.6 (22.6-62.0)** | **62.3 (32.6-77.6)** | **36.5 (21.1-50.1)** | **0.015** |
| Day 28 | 44.4 (29.7-61.1)  (n=38) | 61.9 (27.7-91.9)  (n=6) | 43.8 (30.3-50.1)  (n=32) | 0.317 |

***^a^, P < 0.05; Categorical variables were expressed as n (%); Continuous variables were expressed as Mean ± SD or Median (IQRs); Bold font indicates the difference was statistically significant. CMV: Cytomegalovirus; HRCT: High-resolution Computed Tomography; NT-PCP-III: N-terminal Peptide of Serum Procollagen III.***

**Table S2. Correlation Analysis of Chest HRCT Score and NT-PCP-III.**

| **Variables** | **NT-PCP-III^b^** | **r** | ***P*** |
| --- | --- | --- | --- |
| **Chest HRCT score^a^** | **-** | **0.249** | **0.020** |

***^a^, P < 0.05; ^b^, Day 1; Bold font indicates the difference was statistically significant. Correlation coefficients were calculated using the Spearman correlation coefficient. HRCT: High-resolution Computed Tomography; NT-PCP-III: N-terminal Peptide of Serum Procollagen III; r: Correlation Coefficient.***

**Table S3. Risk Factors for Active CMV I**nfection.

| **Variables** | **β** | **OR (95% CI)** | ***P*** |
| --- | --- | --- | --- |
| **Univariate logistic regression** |  |  |  |
| Platelet^a^ | -0.008 | 0.992 (0.984-1.000) | 0.045 |
| NK cells (%) | -0.171 | 0.843 (0.698-1.017) | 0.075 |
| Blood transfusion^a, b^ | 1.507 | 4.514 (1.209-16.846) | 0.025 |
| Septic shock^a^ | 2.205 | 9.070 (1.126-73.087) | 0.038 |
| **Multivariate logistic regression** |  |  |  |
| Platelet^a^ | -0.009 | 0.991 (0.983-1.000) | 0.042 |
| Septic shock^a^ | 2.208 | 9.097 (1.097-75.453) | 0.041 |

***^a^, P< 0.05; ^b^, Before ICU Admission. CMV: Cytomegalovirus; β: Regression Coefficient; OR: Odds Ratio; CI: Confidence Interval.***

**Table S4. Predictive Value of Platelet on Active CMV** Infection Evaluated by ROC.

|  | **AUC** | **Specificity (%)** | **Sensitivity (%)** | **95% CI** | ***P*** |
| --- | --- | --- | --- | --- | --- |
| Platelet*^a^* | 0.711 | 88.7 | 50.0 | 0.562-0.860 | 0.013 |

***^a^, P< 0.05; CMV: Cytomegalovirus; ROC: Receiver Operating Characteristic; AUC: Area Under the ROC Curve; CI: 95% Confidence Interval.***

**Table S5. Bacterial and Fungal Species.**

| **Species** | **Total** | **Active CMV infection** | | | ***P*** |
| --- | --- | --- | --- | --- | --- |
|  | **N=87** | **Yes**  **(n=14, 16.1%)** | | **No**  **(n=73, 83.9%)** |  |
| **Bacterial Species, n (%)** |  |  |  | |  |
| **Sputum Smear** |  |  |  | |  |
| G^-^ | 75 (86.2) | 13 (92.6) | 62 (84.9) | | 0.715 |
| G^+^ | 46 (52.9) | 10 (71.4) | 36 (49.3) | | 0.129 |
| **Sputum Culture (ETA)** |  |  |  | |  |
| Acinetobacter Baumannii | 35 (40.2) | 7 (50) | 28 (38.4) | | 0.416 |
| Stenotrophomonas Maltophilia | 23 (26.4) | 7 (50) | 16 (21.9) | | 0.064 |
| Klebsiella Pneumoniae | 22 (25.3) | 2 (14.3) | 20 (27.4) | | 0.485 |
| Pseudomonas Aeruginosa | 16 (18.4) | 3 (21.4) | 13 (17.8) | | > 0.999 |
| Burkholderia Cepacia | 9 (10.4) | 3 (21.4) | 6 (8.2) | | 0.314 |
| Escherichia Coli | 4 (4.6) | 1 (7.1) | 3 (4.1) | | 0.511 |
| Enterococcus Faecium | 4 (4.6) | 1 (7.1) | 3 (4.1) | | 0.511 |
| Staphylococcus Haemolyticus | 4 (4.6) | 0 (0) | 4 (5.5) | | > 0.999 |
| Staphylococcus Aureus | 3 (3.5) | 0 (0) | 3 (4.1) | | > 0.999 |
| Corynebacterium Striatum | 2 (2.3) | 0 (0) | 2 (2.7) | | > 0.999 |
| Ralstonia Mannitolilytica | 2 (2.3) | 0 (0) | 2 (2.7) | | > 0.999 |
| H. Influenzae | 2 (2.3) | 0 (0) | 2 (2.7) | | > 0.999 |
| Enterobacter Aerogenes | 2 (2.3) | 0 (0) | 2 (2.7) | | > 0.999 |
| Staphylococcus Epidermidis | 2 (2.3) | 0 (0) | 2 (2.7) | | > 0.999 |
| Negative | 16 (18.4) | 1 (7.1) | 15 (20.6) | | 0.418 |
| **Fungal Species, n (%)** |  |  |  | |  |
| **Sputum Smear** |  |  |  | |  |
| Fungal Mycelium | 9 (10.4) | 2 (14.3) | 7 (9.6) | | 0.960 |
| **Sputum Culture (ETA)** |  |  |  | |  |
| Candida Albicans | 9 (10.4) | 1 (7.1) | 8 (11.0) | | > 0.999 |
| Oidium Tropioale | 6 (6.9) | 1 (7.1) | 5 (6.9) | | > 0.999 |
| Candida Parapsilosis | 5 (5.8) | 2 (14.3) | 3 (4.1) | | 0.181 |
| Trichosporon Sp | 2 (2.3) | 0 (0) | 2 (2.7) | | > 0.999 |
| Saccharomyces Cerevisiae | 1 (1.2) | 1 (7.1) | 0 (0) | | 0.161 |
| Aspergillus Fumigatus | 1 (1.2) | 0 (0) | 1 (1.4) | | > 0.999 |
| Aspergillus Terreus | 1 (1.2) | 1 (7.1) | 0 (0) | | 0.161 |
| Negative | 63 (72.4) | 9 (64.3) | 54 (74.0) | | 0.677 |

***Categorical variables were expressed as n (%); CMV: Cytomegalovirus; ETA: Endotracheal Aspirates; G^+^: Gram-positive; G^-^: Gram-negative.***

**Table S6. Detection Time-point of CMV DNAemia and Chest HRCT Scans.**

|  | **Total**  **N (%)** | **Time** | | | | |
| --- | --- | --- | --- | --- | --- | --- |
|  |  | **D1**  **(%)** | **D2-7 (%)** | **D8-14 (%)** | **D15-21 (%)** | **D22-28 (%)** |
| CMV DNAemia (+) | 14 (100) | 8 (57.2) | 5 (35.7) | 1 (7.1) | 0 (0) | 0 (0) |
| Chest HRCT Scans^ (2^rd^) | 14 (100) | 0 (0) | 1 (7.1) | 2 (14.3) | 2 (14.3) | 9 (64.3) |

***^, CMV DNAemia positive; Categorical variables were expressed as n (%); CMV: Cytomegalovirus; HRCT: High-resolution Computed Tomography.***
